# A Double-Blind, Randomized, Placebo-Controlled Trial to Evaluate the Efficacy of a Hydrolyzed Chicken Collagen Type II Supplement in Alleviating Joint Discomfort

**DOI:** 10.3390/nu13072454

**Published:** 2021-07-18

**Authors:** Anaam Mohammed, Siran He

**Affiliations:** 1 Pro Case Management Trials, Denver, CO 80218, USA; anaam.mohammed@gmail.com; 2 Milken Institute School of Public Health, The George Washington University, Washington, DC 20052, USA

**Keywords:** collagen type II, joint discomfort, nutritional supplement

## Abstract

Joint pain and disease affects more than one in four adults in the United States. We conducted a double-blind, randomized, placebo-controlled trial to investigate the efficacy of a hydrolyzed chicken collagen type II (HCII) supplement in reducing joint-related discomfort such as pain and stiffness, and in improving mobility. We enrolled adults aged 40–65 (65.5% were women) who had joint discomfort, but had no co-morbidities, and who were not taking pain medications. The participants were randomized to receive either the HCII supplement (*n* = 47) or a placebo (*n* = 43) for eight weeks. At the baseline, and at week 4 and week 8, we administered the Western Ontario and McMaster Universities Arthritis Index (WOMAC) survey with three additional wrist-related questions and the Visual Analog Scale for assessments of joint-related symptoms. In the WOMAC stiffness and physical activity domains and in the overall WOMAC score, the HCII group had a significant reduction in joint-related discomforts compared with the placebo group. For example, at week 4, the HCII group had a 36.9% reduction in the overall WOMAC score, compared with a 14.3% reduction in the placebo group (*p* = 0.027). This HCII product is effective in reducing joint pain and stiffness and in improving joint function among otherwise healthy adults.

## 1. Introduction

Arthritis, which refers to joint pain or joint disease, affects at least one in four adults in the United States (US) [1], among whom 23.7 million individuals have reported joint discomfort that causes limitations in activities [1]. The prevalence of arthritis in the US affects approximately 92.1 million people, which may still be an underestimation of the actual burden, particularly in adults under the age of 65 years. Specifically, for adults aged 18–64, almost one in three have physician-diagnosed arthritis or have reported joint symptoms consistent with an arthritis diagnosis. The estimates are worse for those over the age of 65 [2]. There are over 100 types of arthritis, and the most common type is osteoarthritis (OA). A degenerative joint disease, OA is characterized by the progressive deterioration of the articular cartilage [3]. An estimated 30.8 million adults in the US have an OA diagnosis [4]. OA is also the most common cause of disability in adults [3].

Treatments of joint-related pain usually focus on reducing pain, retaining mobility, and minimizing disability. Nonsteroidal anti-inflammatory drugs (NSAIDs) are the most widely used drugs in this patient population [5]. Although NSAIDs can help alleviate pain, they only provide partial symptomatic relief and do not curb underlying disease progression. There are also risks associated with the consumption of NSAIDs, such as gastrointestinal side effects and cardiovascular complications [6].

Collagen products are recognized as safe components of nutraceuticals, pharmaceuticals, and foods by the US Food and Drug Administration (under the Code of Federal Regulation, Title 21 CFR §184.1553), the Center for Food Safety and Nutrition, and the Natural Ingredients and Organic Foods Industry, and have therefore been explored in relation to the treatment of joint discomfort. Of the 28 types of collagen, type II collagen is one of the most common. Approximately 50% of all the protein in the body’s joint cartilage is type II collagen. Orally administered hydrolyzed collagen has been found to be absorbed intestinally and stimulates chondrocytes to produce type II collagen [7,8]. Some scientists therefore suggest that hydrolyzed collagen type II (HCII) may have the potential to repair or regenerate deteriorating collagen [9].

In a review of the studies examining collagen hydrolysate for the treatment of OA and other joint disorders, Bello and Oesser described a German study of 100 participants who suffered from hip, knee, and shoulder pain resulting from intense physical activity [8]. When given 10 g of hydrolyzed collagen daily for 12 weeks, 78% of the participants reported reduced pain; however, the study failed to provide sufficient details regarding the statistical analysis [8]. Another randomized, double-blind study comparing hydrolyzed collagen to a placebo in patients with OA of the knee found that those who took 10 g of collagen daily for 6 months had a significant reduction in pain (measured by the Visual Analogue Scale, or VAS) when compared to those in the placebo group. However, there was no statistically significant difference between the treatment groups in the Western Ontario and McMaster Universities Arthritis Index (WOMAC) score, which represents overall joint health [10]. Schauss and colleagues conducted a randomized, double-blind placebo trial to evaluate BioCell Collagen in the treatment of OA symptoms [11]. The collagen group experienced significant improvements in physical activities compared to the placebo group at 35 days and 70 days. It should be noted, however, that the subjects in this study were allowed to take pain medicine.

Avicenna’s Hydrolyzed Chicken Collagen Type II supplement, AVC-H2, was designed and developed with help from the Rose–Hulman Institute of Technology. In this double-blind, randomized, placebo-controlled trial, we evaluated the efficacy of AVC-H2 among adults with joint discomfort, such as pain, stiffness, and reduced joint mobility. To our knowledge, this is the first study to assess the effects of a HCII supplement on joint symptoms in a population not consuming pain medication. It is also the first study to evaluate the efficacy of HCII on wrist discomfort in addition to knee and hip discomfort.

## 2. Materials and Methods

### 2.1. Study Population and Design

The double-blind, randomized, placebo-controlled trial (ClinicalTrials.gov Identifier: NCT04955353) was conducted in the US to investigate the efficacy of AVC-H2 in alleviating symptoms of joint-related discomfort, such as pain and stiffness, and in improving joint mobility. The study was conducted between October 2017 and February 2018, in compliance with the International Conference of Harmonization Tripartite Guidelines on Good Clinical Practice, applicable FDA regulations/guidelines set forth in 21 CFR Parts 11 and 50, and the standard practices of BioScreen, at a BioScreen Clinical Testing Services, Inc. facility (Phoenix, AZ, USA).

Study participants were recruited through advertisements in local periodicals, community bulletin boards, phone solicitation, electronic media, or any combination thereof. The inclusion criteria were: men and women 40–65 years of age; all races; good general health (free of any systemic or dermatological disorder) but with joint pain, stiffness, or other joint-related discomfort; literate and able to understand the informed consent document, as well as able to choose whether or not to sign the form; able to complete a preliminary medical history, Health Insurance Portability and Accountability Act (HIPAA), and Code of Conduct form; able to cooperate with the investigator and research staff; willing and able to comply with the instructions to use the test product, and to complete the full course of the study; agree to not participate in any other study during the entire duration of the study; have not participated in a similar study in the past thirty days; agree to refrain from using any joint-pain relief products except for the product received from our research staff during the entire duration of the study; agree to take a urine pregnancy test prior to enrollment.

Exclusion criteria were: individuals currently taking any medications that may mask or interfere with the test results; those who had a history of any acute or chronic disease that would interfere with or increase the risk of study participation; those with a history of diseases of chronic inflammation (e.g., septic arthritis, gout, and rheumatoid arthritis); those who had a recent injury in the hip, knee, and/or wrist in the past six months; those who had a history of hip, knee, and/or wrist surgery; those who had injections to reduce joint discomfort in the past 12 months; those who are at high risk of adverse outcomes for participating in the study and thus may invalidate the study due to confounding factors; pregnancy or lactation; individuals who had diabetes or hyperuricemia; body mass index > 30 kg/m^2^; history of substance abuse; known history of hypersensitivity to any cosmetics, personal care products, or fragrances; known allergy to eggs, chicken, or any other ingredients in the test product; individuals who have phenylketonuria; those who need to take calcium supplementation; or those who are affiliated with the clinical trial agency.

Following a standardized computer randomization procedure, enrolled participants who provided informed consent were randomized to receive either AVC-H2 or a placebo. Participants in the AVC-H2 group were instructed to take four pills daily: two pills every morning on an empty stomach, and 30 min before the first meal with a glass of water; and two pills every evening at least two h after the last meal (or before bed) with a glass of water. In the event that the participant forgot to take the morning dosage on an empty stomach, s/he was instructed to wait at least two h after the meal and then consume the morning dosage. Participants in the placebo group were instructed to do exactly the same as in the AVC-H2 group.

All participants were followed up at weeks 4 and 8 after the baseline assessment, and the study was completed within an eight-week period. A total of 90 participants completed the study.

### 2.2. Materials

The product used was Avicenna’s Hydrolyzed Chicken Collagen Type II raw material, AVC-H2, a patented and proprietary formula. The technique used to create AVC-H2 is acid-based hydrolysis, which mimics the body’s natural hydrolysis process for ease of digestion and an optimum low molecular weight. AVC-H2 protein consists of protein bonds that have been broken down or “untied” so that they are more easily absorbed by the small intestines. To produce AVC-H2, a full-frame chicken sternum is used in the hydrolysis process. Because the starting raw material is mostly comprised of type II collagen protein, the total collagen in the final product is ≥70%, making it a highly pure product in comparison to other collagen products that were tested prior to the study. AVC-H2 mixes well with other ingredients, and can be used in a variety of ways, including capsules, multi-collagen mixes, protein shakes and nutritional shakes, protein bars, collagen chews, soup or broths, and in other functional foods. More information about AVC-H2 can be found in the patent claims (please refer to the “Patent” section of the manuscript).

The total collagen content of AVC-H2 was based on certificates of analyses prior to the study. As such, a smaller dosage was administered to patients compared to previous studies of similar products [8,10]. Patients in the study were randomly assigned to receive either 2.5 g of AVC-H2 daily or an equal amount of the placebo.

### 2.3. Assessment of Outcomes

All participants were instructed to answer the following surveys at the baseline (post-enrollment, pre-treatment), and week 4 and week 8 of the study: the WOMAC survey for overall joint discomfort assessment and the VAS for pain assessment [12,13].

#### 2.3.1. WOMAC Survey

The original WOMAC questionnaire has 24 questions in three joint-related domains. The version of the WOMAC survey used in our study included three additional questions, one in each domain, and all were related to wrist discomfort (WOMAC + 3) [14]. Therefore, our version included 27 questions in three joint-related domains: six questions in the pain domain (e.g., “how much pain do you have during the last 48 h walking on a flat surface?”); three questions in the stiffness domain (e.g., “how severe is your stiffness after first awakening in the morning during the last 48 h?”); and 18 questions in the difficulty in physical activities domain (e.g., “think about the difficulty you had in doing the following daily physical activities due to your hip/knee/wrist during the last 48 h—descending stairs”).

For each question, the participants could rate their own discomfort on a Likert scale of 0 to 4: “0” referred to no joint-related discomfort, “1” was mild discomfort, “2” represented moderate discomfort, “3” was severe discomfort, and “4” referred to extreme discomfort for this particular question. Scores were added up in each domain to create three sub-scores, and then summed up (without weights) across all domains to form the WOMAC overall score. We conducted a correlation analysis to ensure that the “WOMAC + 3” survey correlated well with the original WOMAC survey. Correlation coefficients (ρ) were >0.8 in all domains, indicating a good correlation between our version of the extended survey and the original survey.

#### 2.3.2. VAS Scores

The Visual Analog Scale for pain assessment is a well-recognized, widely used, and validated measure for acute and chronic pain. The scores are recorded by marking the approximately level of pain on a 10 cm line that represents the range of pain: from left to right, it ranges from “no pain” to “worst pain”. Each centimeter is translated into one point of the VAS and only those with a VAS score of ≥4 at the baseline were included in the final analysis. One note about the assessment of pain in this study is that we reported both the VAS results and the WOMAC pain domain to provide a comprehensive view of our assessments. The readers, however, are encouraged to prioritize the VAS in terms of pain assessment [15].

#### 2.3.3. Assessment of Other Characteristics

We collected information from the baseline characteristics of the study participants, including age, sex, race, behavioral factors, and selected disease status. Body mass index (BMI) was used for weight categorization [16], and participants with a BMI between 18.5 and 24.9 kg/m^2^ were considered to have a normal weight, those with a BMI between 25.0 and 29.9 kg/m^2^ were overweight, and those whose a BMI of ≥30 kg/m^2^ were considered obese. Hypertension was based on self-reported medical history. Baseline smoking status, alcohol consumption, exercise habits, and duration of joint-related pain were all self-reported.

### 2.4. Compliance and Adverse Events

Compliance was strictly monitored throughout the study. All subjects were followed to assess whether they adhered to the protocol. No participation was terminated due to severe protocol violation, but all other protocol violation was documented (see “sensitivity analysis” in the statistical methods section).

In case of adverse events, the events were meticulously documented by study staff, and the participants could decide whether to continue or withdraw from the study. No severe or life-threatening adverse event occurred. All 11 documented adverse events were mild to moderate. Upon investigation, all 11 participants voluntarily withdrew from the study as per the protocol and were not included in the final analyses.

### 2.5. Statistical Analysis

We conducted all analyses in R version 3.6.0 (R Core Team, Foundation for Statistical Computing, Vienna, Austria) [17]. Statistical significance was set a priori at a *p* value of <0.05. All *p*-values were two-sided. Confidence intervals (CI) were reported at the 95% level, and 90% of the CIs were reported in the table footnote where appropriate.

#### 2.5.1. Intention-to-Treat (ITT) Analysis

We first described the participants based on their assigned group. For comparisons of the baseline characteristics between the AVC-H2 group and the placebo group, we used the Student’s *t*-test for continuous variables, the χ² test for categorical variables, and supplemented the latter with Fisher’s exact test for categorical variables with a low cell count.

We reported both the raw values and the changes from the baseline for the WOMAC overall score and sub-scores, as well as for the VAS. Relative changes from the baseline to the follow-up time points (week 4 or week 8) for each score and sub-score were the main indicators in this study, because they reflect the magnitude of change in the two groups, which enables us to compare not just the raw values but the relative extent of change. Relative change is expressed as %Δ:

(1) $$\frac{Score\;at\;followup\;timepoint-Baseline\;score}{Baseline\;score\;}\times \;100\%$$

Comparisons between the AVC-H2 group and the placebo group included: (i) comparisons of the raw scores at the baseline and each follow-up time point, and (ii) comparisons of %Δ at week 4 or week 8. The Student’s *t*-test for continuous variables with normal distribution and the Mann–Whitney U test for continuous variables with non-normal distribution were used. We also conducted a least squares regression analysis for %Δ comparisons, and the formula is:

*Y_i_* = *β_0_* + *β_1_X_i_* + *ε_i_* (2)

In this formula, *Y* represents the dependent variable (joint-related discomfort, assessed through WOMAC and VAS), *X* is the independent variable (product, AVC-H2 versus placebo), and *β_1_* is the key measurement, for which the study calculated confidence intervals. *β_0_* denotes the intercept, whereas *ε_i_* is the random error component, and we did not report these two components.

Outliers and implausible values were excluded in each analysis based on both statistical and clinical rationales. For instance, if a participant had 0 as a WOMAC baseline score, the %Δ is incalculable, and would thus be excluded by default. In this case, there would not be any improvements in joint-related discomfort in subsequent weeks, thus rendering the value clinically meaningless as well. In a few other cases, empirical clinical knowledge was applied to assess the biological and clinical plausibility of a value. Eventually, between 0 and 3 participants were removed in each set of analyses, which is reported in the footnote of each table.

Besides the main analyses described above, we also conducted a sub-group analysis by stratifying the participants based on the type of discomfort (injury-related or otherwise; severe or not severe discomfort at baseline), sex, weight categories, smoking status, alcohol consumption, and exercise at the baseline. In addition, the study also tested the interaction term between age and test product, as well as between the duration of pain prior to enrollment in the study and the test product. For the VAS, we reported the number of participants who had a major improvement in pain, which included participants whose VAS score reduced by three or more points from the baseline to each follow-up time point. The study then compared the proportion of participants that fell in this category between the two groups.

#### 2.5.2. Sensitivity Analysis

To further test the robustness of the main analysis, 13 non-compliant participants were removed, three in the AVC-H2 group and ten in the placebo group. The reasons were for protocol violations, which included two participants using over-the-counter pain medication, and 11 participants taking calcium supplementation during the study. The same analyses were conducted as described in the ITT section.

### 2.6. Data Availability

The data presented in this study are not available due to intellectual property rights.

### 2.7. Research Ethics

Institutional review board (IRB) approval was granted by Chesapeake IRB (now part of Advarra^®^, Columbia, MD, USA) in October 2017. The Approved Protocol Number is BCS 17–033; the IRB Approval Number is MOD00232293. Participation in this study was voluntary, and all participants provided written consent (signed and dated) prior to enrollment in the study to indicate that they were informed of the reasons for the study, possible adverse effects, associated risks and potential benefits, as well as their limits of liability.

## 3. Results

### 3.1. Baseline Characteristics of the Study Participants

Out of the 90 participants who were included in the study, 47 were in the AVC-H2 group and 43 were in the placebo group, and all were within similar age ranges (Table 1). Over half of the participants were women (61.7% in the AVC-H2 group and 69.8% in the placebo group). The distribution of race was similar between the two groups (*p* = 0.951).

Regarding health conditions and risk factors at the baseline, most characteristics were not statistically significantly different between the two groups (*p* ≥ 0.05), except the duration of pain reported at the beginning of the study (Table 1). On average, the AVC-H2 group reported a significantly longer duration of joint-related pain than the placebo group (*p* = 0.023). The BMI values were similar between the two groups (*p* = 0.197). Nevertheless, standardized categorization showed that 53.2% of the AVC-H2 group and 44.2% of the placebo group participants were overweight, and 8.5% (AVC-H2) and 4.7% (placebo) were obese. Hypertension affected approximately one in ten participants (12.8% in the AVC-H2 group and 9.3% in the placebo group, *p* = 0.742). About 20% of the participants reported smoking at the baseline, and between 30 and 50% of all participants reported alcohol consumption at the baseline.

### 3.2. WOMAC Findings

At the baseline, the WOMAC overall score and sub-scores in the pain, stiffness, and difficulty in physical activity domains were similar between the two groups (*p* > 0.05 for all baseline raw score comparisons) (Table 2).

**Figure 1:**
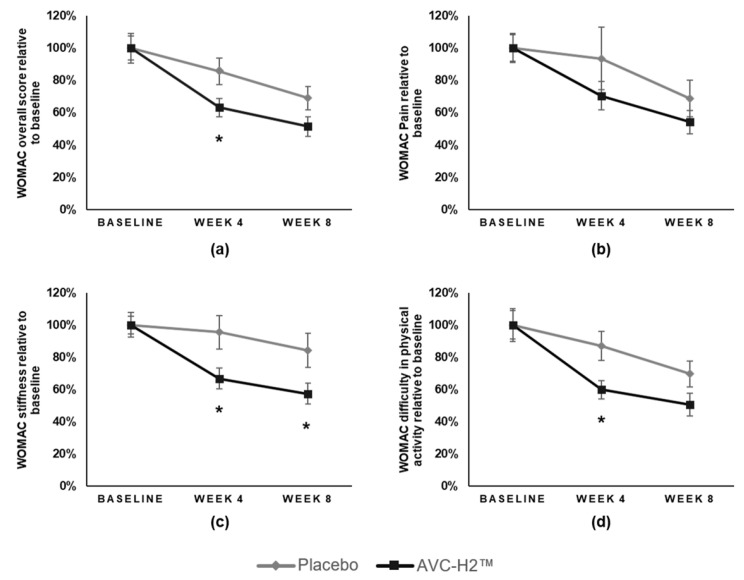
Relative changes in the WOMAC scores by intervention group. The graphs represented the relative change in scores from the baseline to the follow-up weeks: (**a**) WOMAC overall score; (**b**) WOMAC pain domain; (**c**) WOMAC stiffness domain; (**d**) WOMAC difficulty in physical activity domain. Baseline scores were standardized as 100% across domains for comparison purposes. Error bars represented the standard error (SE) for each group at different time points in each specific WOMAC domain. * *p* < 0.05 for between-group comparisons at a given follow-up time point. Abbreviations: AVC-H2, Avicenna’s Hydrolyzed Chicken Collagen Type II; WOMAC, The Western Ontario and McMaster Universities Arthritis Index.

At week 4, the WOMAC overall score reduced by 36.9% in the AVC-H2 group, which was a value significantly larger in magnitude than the 14.3% reduction in the placebo group (β = −22.6; 95% CI, −40.2 to −3.3; *p* = 0.027). At week 8, the WOMAC overall score reduced by 48.6% in the AVC-H2 group and 31.0% in the placebo group, and the differences in %Δ were statistically significant at 90% CI (β = −17.7; 90% CI, −33.3 to −2.1; *p* = 0.065) (Table 2, Figure 1a).

In the WOMAC pain domain at week 4, the AVC-H2 group had a reduction of 29.5%, whereas the placebo group had a 6.5% reduction; at week 8, the AVC-H2 group had a 45.8% reduction, and the placebo group had a 31.4% reduction. Despite the observable differences between the two groups, with more pain reduction in the AVC-H2 group than in the placebo group, the differences were not significant at both follow-up time points (Table 3, Figure 1b).

The WOMAC sub-score in the stiffness domain had significant between-group differences at week 4 and week 8, both favoring the AVC-H2 group. At week 4, the AVC-H2 group had a 33.2% reduction in stiffness, whereas the placebo group had a 4.5% reduction (β = −28.6; 95% CI, −52.3 to −5.0; *p* = 0.022); at week 8, the AVC-H2 group had a 42.6% reduction in stiffness and the placebo group had a 15.7% reduction (β = −26.9; 95% CI, −51.0 to −2.9; *p* = 0.034) (Table 4, Figure 1c).

Similar to the WOMAC overall score, the change in the WOMAC sub-score in the difficulty in physical activities domain had a statistically significant difference at week 4, with a 40.2% reduction in the AVC-H2 group and a 12.9% reduction in the placebo group (β = −27.3; 95% CI, −48.0 to −6.6; *p* = 0.014). There was also a significant difference at a 90% CI at week 8, with a 49.4% reduction in the AVC-H2 group and a 30.4% reduction in the placebo group (β = −19.0; 90% CI, −36.8 to -1.3; *p* = 0.079) (Table 5, Figure 1d).

Qualitatively, in all the WOMAC domains (and in the overall score), the AVC-H2 group had more improvements in reducing joint-related discomforts than in the placebo group. Figure 1 illustrates this point through the similar trend line of symptom relief in all domains. The line (black) representing the AVC-H2 group is consistently below the line (gray) representing the placebo group (Figure 1a–d).

### 3.3. VAS Findings

**Figure 2:**
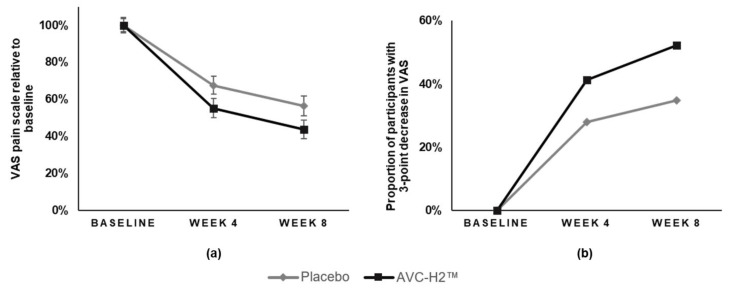
Relative changes in the VAS pain assessment scale and the proportion of participants with a 3-point pain reduction, by intervention group. (**a**) relative change in VAS from the baseline to the follow-up weeks. Baseline VAS was standardized as 100%; (**b**) the proportion of participants who had ≥3 points decrease in the VAS pain scale from the baseline to the follow-up weeks. Error bars represent the standard error (SE) for each group at different time points in VAS. Abbreviation: AVC-H2, Avicenna’s Hydrolyzed Chicken Collagen Type II; VAS, Visual Analog Scale.

At the baseline, the AVC-H2 group and the placebo group had similar VAS raw scores (*p* > 0.05) (Table 6). The AVC-H2 group had a larger reduction in pain based on the VAS at both week 4 and week 8, compared with the placebo group. Both differences were statistically significant at 90% CI: at week 4, the AVC-H2 group had a 44.8% reduction in pain, and the placebo group had a 32.4% reduction (β = −12.4; 90% CI, −24.0 to −0.7; *p* = 0.079); at week 8, the AVC-H2 group had a 56.2% reduction in pain, compared to a 42.7% reduction in the placebo group (β = −12.4; 90% CI, −24.5 to −0.3; *p* = 0.092) (Table 6, Figure 2a).

It was also observed that for participants with greater decreases in VAS scores (reduction of three or more points from the baseline to the follow-up weeks), there was a difference between the two groups (Figure 2b), with major differences shown in the AVC-H2 group versus the placebo group. At week 4, 41% of the participants in the AVC-H2 group had a major reduction in pain versus 28% of participants in the placebo group. At week 8, 52% of participants in the AVC-H2 group had a major reduction in pain versus 35% of participants in the placebo group (Figure 2b, exact data not shown).

### 3.4. Additional Findings

**Figure 3:**
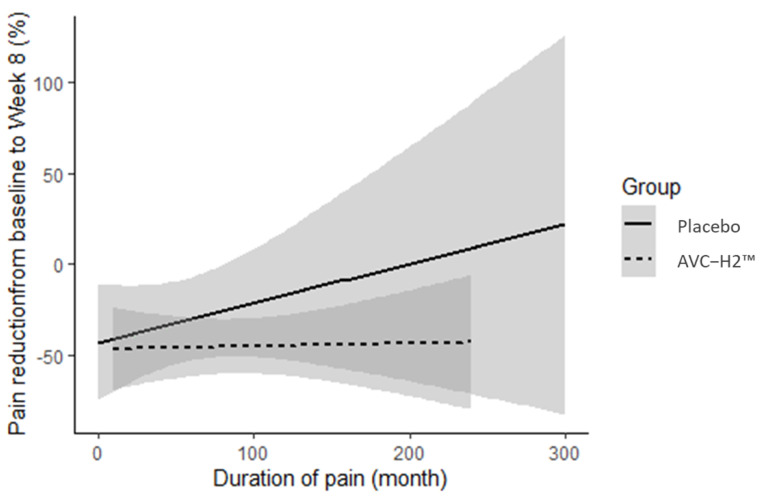
Pain reduction from the baseline to week 8 by duration of pain in the two groups. Duration of pain in months refers to how long the participants had experienced joint-related pain prior to enrollment in our study. Abbreviation: AVC-H2, Avicenna’s Hydrolyzed Chicken Collagen Type II.

The reduction in pain in the AVC-H2 group was consistent based on the WOMAC sub-score in the pain domain, regardless of the duration of pain prior to study enrollment. That is, a reduction in pain was observed for both individuals who had been suffering from pain for years and those who had been suffering for only months or weeks. This was unlike in the placebo group, where the pain reduction was abated when the pain was more chronic (longer duration prior to study) (Figure 3).

Several additional findings are worth noting. In the WOMAC stiffness domain, it was observed that the participants who reported non-severe stiffness at the baseline (reported 0 to 2 in any stiffness question) further benefited from AVC-H2, with a starker between-group difference in %Δ. Among the participants with severe stiffness at the baseline (reported 3 or 4 in any stiffness question), the outcome was β = −5.7 (95% CI, −36.1, 24.8; *p* = 0.718), whereas in participants who reported non-severe stiffness, the outcome was β = −34.9 (95% CI, −66.0 to −3.8; *p* = 0.032) (Supplemental Table S1).

### 3.5. Sensivitity Analysis Findings

Based on the sensitivity analysis, after removing the non-compliant participants, the main findings in the WOMAC and the VAS were not meaningfully altered. Similar patterns were also observed in the WOMAC overall score and the sub domains as well as in the VAS, which confirmed the beneficial effect of AVC-H2 in reducing joint-related discomforts compared with the placebo group.

Specifically, we observed a significantly larger reduction in the WOMAC overall score in the AVC-H2 group than in the placebo group at week 4 (β = −21.8; 95% CI, −41.9 to −1.7; *p* = 0.045), and observable differences at week 8 in favor of the AVC-H2 group (Supplemental Table S2). It was observed that there was not a statistically significant between-group difference in the reduction in the WOMAC pain sub-score at both week 4 and week 8 (Supplemental Table S3). However, it was confirmed that the most statistical significance was observed in the WOMAC stiffness domain, favoring the AVC-H2 group at both week 4 (β = −27.3; 95% CI, −48.6 to −6.0; *p* = 0.018) and week 8 (β = −23.1; 95% CI, −45.0 to −1.1; *p* = 0.048) (Supplemental Table S4). For the WOMAC sub-score in the difficulty in physical activities domain, there was a larger reduction in the AVC-H2 group than in the placebo group, which was significant at week 4 (β = −27.1; 95% CI, −49.3 to −4.9; *p* = 0.029), and observable differences at week 8 in favor of the AVC-H2 group (Supplemental Table S5).

Similar to the study’s main findings, the same pattern was observed in VAS pain reduction in the sensitivity analysis, favoring the AVC-H2 group at both week 4 and week 8 with significance at 90% CI (week 4, β = −13.3; 90% CI, −26.3 to −0.2; *p* = 0.090; week 8, β = −14.8; 90% CI, −28.4 to −1.1; *p* = 0.071) (Supplemental Table S6). One final note: the difference in the *p*-value at week 8 in the WOMAC overall score and at week 8 in the physical activity domain score may be attributed to a further decreased sample size in the sensitivity analysis.

## 4. Discussion

Daily intake of the hydrolyzed collagen type II supplement (AVC-H2) for eight weeks resulted in a significant reduction in joint pain and stiffness and increased mobility. AVC-H2 was effective in reducing VAS-assessed pain among participants who consumed 2.5 g of AVC-H2 daily. A significantly larger improvement was observed in the overall WOMAC +3 score and in the stiffness and activity sub-domains of WOMAC in the AVC-H2 group, as compared with the placebo group. Both the intention-to-treat analysis and the sensitivity analysis that removed non-compliant participants yielded similar results.

A similar improvement was also observed in joint discomfort attributable to the intake of the HCII product as previously reported [11]. Another study conducted by Kumar and colleagues also reported that the ingestion of collagen hydrolysate improved the WOMAC score in 30 participants with diagnosed knee osteoarthritis [18]. Nevertheless, the Kumar et al. study differed from our current study in two key ways: first, we excluded patients with known osteoarthritis, because our goal was to examine the effect of AVC-H2 in a general population; second, during the placebo run-in period, Kumar et al. allowed the patients to receive baseline therapy of aceclofenac, which is a type of NSAIDs [18]. To our knowledge, this study is the first to assess the effect of HCII on joint discomfort among adults not utilizing any pain medication. This distinction is important because NSAIDs, analgesics, or other supplements could confound the effect of HCII on joint function and pain. Previous studies evaluating the effects of hydrolyzed collagen on joint discomfort allowed participants to simultaneously take NSAIDs, analgesics, antipyretics, COX−2 inhibitors, and/or corticosteroids, making it unclear whether the reduction in joint discomfort was due to the medications or the collagen [8,11].

This study also appears to be the first to assess joint discomfort in the wrists in addition to other commonly assessed joints such as the hip and knee [10,11]. We observed that the reduction in pain in the AVC-H2 group was consistent, regardless of how long participants had experienced pain prior to study enrollment. In the placebo group, conversely, pain reduction was abated when the pain was more chronic at the baseline.

In a previous study, Benito-Ruiz and colleagues administered 10 g/day of hydrolyzed porcine collagen to the study participants [10]. In contrast, a much smaller dose of AVC-H2 (2.5 g/day) was administered to the participants in our study. Despite the modesty in the dosing regime in our study, we observed a significant reduction in joint discomfort, as characterized by the WOMAC survey. This level of significant reduction was not noted in the WOMAC Survey in the Benito-Ruiz study [10]. Interestingly, in the Benito-Ruiz study, only those who did not eat meat habitually showed significant improvements in the VAS. It is possible that the lack of a significant reduction in the WOMAC when consuming 10 g of hydrolyzed porcine collagen is due to the fact that hydrolyzed porcine collagen contains more type I and III proteins, which are essential for hair, skin, and nails, and may not have targeted effects on the joints. It is important to note that the AVC-H2 product is mostly derived from chicken cartilage, which includes the sternum cartilage. Chicken collagen is known to contain more of the type II protein than porcine collagen, which is mostly derived from the hide or bones of the animal and known to contain more type I and III proteins [10,19,20,21].

It was also observed that there was a more significant reduction in joint stiffness and increased mobility in the AVC-H2 group compared with the placebo group. This may be explained by the process through which hydrolyzed collagen is absorbed intestinally, accumulates in the cartilage, and stimulates the regeneration of chondrocytes. That is, AVC-H2 is suspected to target the underlying pathology of joint stiffness. Joint pain, a potential symptom of cartilage degradation, is thus addressed by the hydrolyzed collagen type II supplement [8].

It is important to note that the inclusion criteria of our study were broader than those of previous studies. We included participants who were of good general health but who reported joint pain, stiffness, or other joint-related discomfort, as opposed to those specifically with an OA diagnosis [10,11]. As such, our findings may be more generalizable to adults in the US experiencing any type of arthritis, not just OA.

In this study, a total of 13 participants were non-compliant, three in the AVC-H2 group and ten in the placebo group; two participants used an over-the-counter pain medication and 11 took calcium supplements during the study. These participants were removed from the data during the sensitivity analysis, and the main analysis results were confirmed. Removing them from the analysis still yielded a significant reduction in joint discomfort among the AVC-H2 group, as compared with the placebo group.

Important limitations of this study include the relatively small sample size and the study duration of eight weeks. Additional studies are necessary to further evaluate the long-term effects of AVC-H2 consumption. Assessing additional clinical measures, instead of relying solely on self-reported information, will also provide a more robust understanding of the effects of AVC-H2 on overall joint discomfort. These measures include but are not limited to additional wrist measures and the assessment of other joint functions, such as the shoulder or ankle. A longer study duration would allow for a further investigation into the effects of AVC-H2 on joint pain and other associated discomfort and functional outcomes. In addition, we may not have been able to capture sub-clinical changes in terms of inflammatory and pathophysiological progression of joint functions through WOMAC and VAS assessments. Future studies should include relevant biomarkers (such as interleukin−6, C-reactive protein, and other sensitive inflammation markers) to investigate any potential biochemical disturbances [22].

This study has several strengths, including the use of a HCII product with a ≥70% total collagen content and a rigorous double-blind randomized placebo-controlled trial study design. The main statistical methods and analyses were robust, which were further confirmed by a sensitivity analysis. In addition, because the study population included those with general joint pain, stiffness, and lack of mobility, rather than diagnosed OA patients alone, the findings may have broader implications for AVC-H2′s effects in reducing joint discomfort among the general population. Future research is warranted to confirm this postulation.

## 5. Conclusions

In this study, adults with joint-related discomfort, who were otherwise healthy and did not consume pain medication for the duration of the study, were randomized to receive either the AVC-H2, a hydrolyzed chicken collagen type II supplement, or an identical placebo. Findings of this study show that AVC-H2 is effective in reducing joint pain and stiffness, and in improving mobility. We also observed that it is a safe nutra-pharmaceutical for use by adults suffering from arthritis and other joint discomfort.

## 6. Patents

The AVC-H2 product reported in this manuscript is patented (Patent Number: US10253090B2) [23].

## Figures and Tables

**Table 1:** Characteristics of the study participants at the baseline.

Characteristics ^1^	Placebo	AVC-H2	*p*-Value ^2^
	(*n* = 43)	(*n* = 47)	
Female, %	69.8	61.7	0.560
Age, year	55.3 ± 5.8	52.1 ± 6.6	0.015
Race, %			0.951
African American	16.3	19.6	
Asian	4.7	6.5	
Caucasian	55.8	52.2	
Hispanic	23.3	21.7	
Body mass index (BMI), kg/m^2^	24.4 ± 3.4	25.3 ± 3.3	0.197
Weight categories, %			0.425
Normal weight	51.2	38.3	
Overweight	44.2	53.2	
Obese	4.7	8.5	
Hypertension, %	9.3	12.8	0.742
Reported smoking, %	20.9	19.1	1000
Reported alcohol consumption, %	32.6	51.1	0.118
Reported regular exercise, %	69.8	74.5	0.794
How long since having the pain, month	56.7 ± 58.4	93.4 ± 89.0	0.023

^1^ Data were presented as mean ± SD or % (when indicated); ^2^ *p*-values for comparisons between two intervention arms, AVC-H2 versus placebo, which were based on: Student’s *t*-test for continuous variables; Chi-squared test for categorical variables; and Fisher’s exact test for categorical variables with a low cell count. A two-tailed *p*-value a prior was set at 0.05; Abbreviations: AVC-H2, Avicenna’s Hydrolyzed Chicken Collagen Type II; SD, standard deviation.

**Table 2:** WOMAC overall scores from the baseline to the follow-up weeks.

Time Point	Measurement ^1^	Placebo ^2^	AVC-H2 ^2^	β Coefficient ^3^	*p*-Value ^4^
		(Mean ± SD)	(Mean ± SD)	(95% CI)	
Baseline	Raw score	30.9 ± 18.5	31.0 ± 15.9	---	0.975
Week 4	Raw score	24.3 ± 17.9	20.3 ± 15.4	---	0.261
	%Δ	−14.3 ± 53.0	−36.9 ± 39.4	−22.6 (−42.0, −3.3)	0.027
Week 8	Score	20.2 ± 18.1	16.3 ± 15.1	---	0.278
	%Δ	−31.0 ± 46.4	−48.6 ± 41.6	−17.7 (−36.1, 0.7) ^5^	0.065

^1^ Raw score obtained from the WOMAC questionnaire; %Δ calculated as the difference between the baseline and the follow-up score, divided by the baseline score, and presented in percentage format. ^2^ Two participants were removed from this set of analysis, 1 in each group, both due to an implausible %Δ value. ^3^ β Coefficient and confidence intervals were obtained from la east squares regression analysis; the values were the AVC-H2 group minus the placebo group. ^4^ *p*-value was based on one of the following methods, where appropriate: Student’s *t*-test for continuous variables with normal distribution; and the Mann–Whitney U test for continuous variables with non-normal distribution. Two-tailed *p*-value a prior was set at 0.05. ^5^ Significant at 90% CI level (−33.3, −2.1). Abbreviations: AVC-H2, Avicenna’s Hydrolyzed Chicken Collagen Type II; CI, confidence interval; SD, standard deviation.

**Table 3:** WOMAC sub-score in the pain domain from the baseline to the follow-up weeks.

Time Point	Measurement ^1^	Placebo ^2^	AVC-H2 ^2^	β Coefficient ^3^	*p*-Value ^4^
		(Mean ± SD)	(Mean ± SD)	(95% CI)	
Baseline	Raw score	7.0 ± 4.1	6.4 ± 3.6	---	0.300
Week 4	Raw score	4.9 ± 3.8	4.3 ± 3.4	---	0.419
	%Δ	−6.5 ± 125.3	−29.5 ± 58.8	−23.0 (−63.3, 17.3)	0.283
Week 8	Score	4.1 ± 4.1	3.4 ± 3.3	---	0.362
	%Δ	−31.4 ± 73.3	−45.8 ± 48.7	−14.4 (−40.2, 11.4)	0.283

^1^ Raw score obtained from the WOMAC questionnaire; %Δ calculated as the difference between the baseline and the follow-up score, divided by the baseline score, and presented in percentage format. ^2^ Two participants were removed from this set of analysis, 1 in each group, both due to incalculable %Δ (baseline score was 0 in this domain). ^3^ β Coefficient and confidence intervals obtained from a least squares regression analysis; the values were the AVC-H2 group minus the placebo group. ^4^ *p*-value was based on one of the following methods, where appropriate: Student’s *t*-test for continuous variables with normal distribution; Mann–Whitney U test for continuous variables with non-normal distribution. A two-tailed *p*-value *a prior* was set at 0.05. Abbreviations: AVC-H2, Avicenna’s Hydrolyzed Chicken Collagen Type II; CI, confidence interval; SD, standard deviation.

**Table 4:** WOMAC sub-score in the stiffness domain from the baseline to the follow-up weeks.

Time Point	Measurement ^1^	Placebo ^2^	AVC-H2 ^2^	β Coefficient ^3^	*p*-Value ^4^
		(Mean ± SD)	(Mean ± SD)	(95% CI)	
Baseline	Raw score	4.4 ± 2.2	4.9 ± 1.8	---	0.243
Week 4	Raw score	3.7 ± 2.3	3.2 ± 2.1	---	0.263
	%Δ	−4.5 ± 68.1	−33.2 ± 44.1	−28.6 (−52.3, −5.0)	0.022
Week 8	Score	3.1 ± 2.1	2.7 ± 2.0	---	0.274
	%Δ	−15.7 ± 69.8	−42.6 ± 44.0	−26.9 (−51.0, −2.9)	0.034

^1^ Raw score obtained from the WOMAC questionnaire; %Δ calculated as the difference between the baseline and the follow-up score, divided by the baseline score, and presented in percentage format. ^2^ One participant was removed from this set of analysis in the AVC-H2 group, due to an incalculable %Δ (baseline score was 0 in this domain). ^3^ β Coefficient and confidence intervals obtained from a least squares regression analysis; the values were the AVC-H2 group minus the placebo group. ^4^ *p*-value was based on one of the following methods, where appropriate: Student’s *t*-test for continuous variables with normal distribution; Mann–Whitney U test for continuous variables with non-normal distribution. A two-tailed *p*-value *a prior* was set at 0.05. Abbreviations: AVC-H2, Avicenna’s Hydrolyzed Chicken Collagen Type II; CI, confidence interval; SD, standard deviation.

**Table 5:** WOMAC sub-score in the difficulty in physical activities domain from the baseline to the follow-up weeks.

Time Point	Measurement ^1^	Placebo ^2^	AVC-H2 ^2^	β Coefficient ^3^	*p*-Value ^4^
		(Mean ± SD)	(Mean ± SD)	(95% CI)	
Baseline	Raw score	20.1 ± 13.0	19.8 ± 11.7	---	0.934
Week 4	Raw score	16.0 ± 12.9	12.8 ± 11.0	---	0.220
	%Δ	−12.9 ± 58.0	−40.2 ± 39.7	−27.3 (−48.0, −6.6)	0.014
Week 8	Score	13.1 ± 12.9	10.2 ± 10.4	---	0.259
	%Δ	−30.4 ± 51.7	−49.4 ± 47.8	−19.0 (−39.9, 1.9) ^5^	0.079

^1^ Raw score obtained from the WOMAC questionnaire; %Δ calculated as the difference between the baseline and the follow-up score, divided by the baseline score, and presented in percentage format. ^2^ Three participants were removed from this set of analysis, 1 in the AVC-H2 group and 2 in the placebo group, due to an implausible %Δ value and an incalculable %Δ (baseline score was 0 in this domain). ^3^ β Coefficient and confidence intervals obtained from a least squares regression analysis; the values were the AVC-H2 group minus the placebo group. ^4^ *p*-value was based on one of the following methods, where appropriate: Student’s *t*-test for continuous variables with normal distribution; Mann–Whitney U test for continuous variables with non-normal distribution. A two-tailed *p*-value *a prior* was set at 0.05. ^5^ Significant at a 90% CI level (−36.8, −1.3). Abbreviations: AVC-H2, Avicenna’s Hydrolyzed Chicken Collagen Type II; CI, confidence interval; SD, standard deviation.

**Table 6:** VAS pain assessment scale from the baseline to the follow-up weeks.

Time Point	Measurement ^1^	Placebo ^2^	AVC-H2 ^2^	β Coefficient ^3^	*p*-Value ^4^
		(Mean ± SD)	(Mean ± SD)	(95% CI)	
Baseline	Raw score	5.2 ± 1.4	5.4 ± 1.3	---	0.624
Week 4	Raw score	3.6 ± 2.1	2.8 ± 1.8	---	0.058
	%Δ	−32.4 ± 30.9	−44.8 ± 34.8	−12.4 (−26.1, 1.3) ^5^	0.079
Week 8	Score	2.9 ± 2.2	2.2 ± 1.8	---	0.091
	%Δ	−43.7 ± 35.0	−56.2 ± 33.6	−12.4 (−26.7, 1.8) ^6^	0.092

^1^ Raw score obtained from the VAS; %Δ calculated as the difference between the baseline and the follow-up score, divided by the baseline score, and presented in percentage format. ^2^ One participant was removed from this set of analysis in the AVC-H2 group due to an implausible %Δ value. ^3^ β Coefficient and confidence intervals obtained from a least squares regression analysis; the values were the AVC-H2 group minus the placebo group. ^4^ *p*-value was based on one of the following methods, where appropriate: Student’s *t*-test for continuous variables with normal distribution; Mann–Whitney U test for continuous variables with non-normal distribution. A two-tailed *p*-value *a prior* was set at 0.05. ^5^ Significant at 90% CI level (−24.0, −0.7). ^6^ Significant at 90% CI level (−24.5, −0.3). Abbreviations: AVC-H2, Avicenna’s Hydrolyzed Chicken Collagen Type II; CI, confidence interval; SD, standard deviation; VAS, Visual Analog Scale.

## Data Availability

The data presented in this study are not available due to intellectual property rights.

## Supplementary Materials

The following are available online at https://www.mdpi.com/article/10.3390/nu13072454/s1, Table S1: WOMAC sub-score change at week 8 in the stiffness domain, stratified by the severity of stiffness at the baseline, Table S2: WOMAC overall score from the baseline to the follow-up weeks, after removing non-compliant participants, Table S3: WOMAC sub-score in the pain domain from the baseline to the follow-up weeks, after removing non-compliant participants, Table S4: WOMAC sub-score in the stiffness domain from the baseline to the follow-up weeks, after removing non-compliant participants, Table S5: WOMAC sub-score in the difficulty in physical activities domain from the baseline to the follow-up weeks, after removing non-compliant participants, Table S6: VAS pain assessment scale from the baseline to the follow-up weeks, after removing non-compliant participants.
