# Supplementary material for: A Double-Blind, Randomized, Placebo-Controlled Trial to Evaluate the Efficacy of a Hydrolyzed Chicken Collagen Type II Supplement in Alleviating Joint Discomfort
Source: Nutrients. 2021 Jul 18;13(7):2454. doi: 10.3390/nu13072454 (PMC8308696; doi:10.3390/nu13072454)
Supplement: Supplementary file 1 [file nutrients-13-02454-s001.zip › nutrients-1257556-supplementary.pdf]

A double-blind, randomized, placebo-controlled trial to evaluate the efficacy of a hydrolyzed chicken collagen type II supplement in alleviating joint discomfort

Mohammed A. and He S.

Supplementary Materials

Table S1. WOMAC sub-score change at week 8 in the stiffness domain, stratified by the severity of stiffness at baseline.

**Baseline stiffness** [1,2]	**β Coefficient** [3]	**(90% CI)**	**(95% CI)**	**p-value** [4]
Pooled (n = 89)	-26.9	(-47.3, -6.5)	(-51.0, -2.9)	0.031
Severe (n = 26)	-5.7	(-32.3, 20.9)	(-36.1, 24.8)	0.718
Non-severe (n = 63)	-34.9	(-61.4, -8.4)	(-66.0, -3.8)	0.032

[1] Baseline stiffness dichotomization: within the stiffness domain, if the participant answered severe (3) or extreme (4) to any of the questions were considered having "severe" stiffness at baseline, otherwise non-severe

[2] For this set of analysis, no outlier was removed

[3] β Coefficient and confidence intervals obtained from least squares regression analysis; the values were AVC-H2 group minus placebo group.

[4] P-value was based on *F*-test in linear regression models. Two-tailed p-value *a prior* set at 0.05

Abbreviations: AVC-H2, Avicenna's Hydrolyzed Chicken Collagen Type II; CI, confidence interval; WOMAC, The Western Ontario and McMaster Universities Arthritis Index survey

Table S2. WOMAC overall score from baseline to follow-up weeks, after removing non-compliant participants.

Time point	Measurement [1]	Placebo [2] (mean ± SD)	AVC-H2 [2] (mean ± SD)	β Coefficient [3] (95% CI)	p-value [4]
Baseline	Raw score	30.1 ± 17.7	29.6 ± 16.2	---	0.889
Week 4	Raw score	25.2 ± 19.8	19.7 ± 15.3	---	0.185
	%Δ	-16.2 ± 51.3	-38.0 ± 38.1	-21.8 (-41.9, -1.7)	0.045
Week 8	Score	20.4 ± 19.5	16.3 ± 15.1	---	0.318
	%Δ	-33.2 ± 46.5	-48.0 ± 41.7	-14.8 (-34.7, 5.1)	0.155

[1] Raw score obtained from the WOMAC questionnaire; %Δ calculated as the difference between baseline and follow-up score, divided by baseline score, and presented in percentage format.

[2] Two participants removed from this set of analysis, 1 in each group, both due to implausible %Δ value.

[3] β Coefficient and confidence intervals obtained from least squares regression analysis; the values were HC group minus placebo group.

[4] P-value was based on one of the following methods, where appropriate: Student's *t*-test for continuous variables with normal distribution; Mann Whitney *U* test for continuous variables with non-normal distribution. Two-tailed p-value *a prior* set at 0.05

Abbreviations: AVC-H2, Avicenna's Hydrolyzed Chicken Collagen Type II; CI, confidence interval; SD, standard deviation; WOMAC, The Western Ontario and McMaster Universities Arthritis Index survey

Table S3. WOMAC sub-score in the pain domain from baseline to follow-up weeks, after removing non-compliant participants.

Time point	Measurement [1]	Placebo [2] (mean ± SD)	AVC-H2 [2] (mean ± SD)	β Coefficient [3] (95% CI)	p-value [4]
Baseline	Raw score	6.8 ± 3.9	6.1 ± 3.6	---	0.422
Week 4	Raw score	5.1 ± 4.1	4.2 ± 3.4	---	0.301
	%Δ	-13.9 ± 60.4	-31.8 ± 57.7	-12.5 (-39.2, 14.2)	0.366
Week 8	Score	4.4 ± 4.3	3.3 ± 3.3	---	0.258
	%Δ	-33.1 ± 54.7	-45.9 ± 48.4	-12.8 (-36.1, 10.4)	0.291

[1] Raw score obtained from the WOMAC questionnaire; %Δ calculated as the difference between baseline and follow-up score, divided by baseline score, and presented in percentage format.

[2] Two participants removed from this set of analysis, 1 in each group, both due to incalculable %Δ (baseline score was 0 in this domain)

[3] β Coefficient and confidence intervals obtained from least squares regression analysis; the values were collagen group minus placebo group.

[4] P-value was based on one of the following methods, where appropriate: Student's *t*-test for continuous variables with normal distribution; Mann Whitney U test for continuous variables with non-normal distribution. Two-tailed p-value *a prior* set at 0.05

Abbreviations: AVC-H2, Avicenna's Hydrolyzed Chicken Collagen Type II; CI, confidence interval; SD, standard deviation; WOMAC, The Western Ontario and McMaster Universities Arthritis Index survey

Table S4. WOMAC sub-score in the stiffness domain from baseline to follow-up weeks, after removing non-compliant participants.

Time point	Measurement [1]	Placebo [2] (mean ± SD)	AVC-H2 [2] (mean ± SD)	β Coefficient [3] (95% CI)	p-value [4]
Baseline	Raw score	4.3 ± 2.0	4.8 ± 1.8	---	0.262
Week 4	Raw score	3.8 ± 2.4	3.1 ± 2.1	---	0.200
	%Δ	-9.6 ± 52.5	-36.9 ± 42.4	-27.3 (-48.6, -6.0)	0.018
Week 8	Score	3.2 ± 2.4	2.6 ± 2.1	---	0.293
	%Δ	-21.3 ± 52.9	-44.4 ± 44.5	-23.1 (-45.0, -1.1)	0.048

[1] Raw score obtained from the WOMAC questionnaire; %Δ calculated as the difference between baseline and follow-up score, divided by baseline score, and presented in percentage format.

[2] One participant removed from this set of analysis in HC group, due to incalculable %Δ (baseline score was 0 in this domain).

[3] β Coefficient and confidence intervals obtained from least squares regression analysis; the values were HC group minus placebo group.

[4] P-value was based on one of the following methods, where appropriate: Student's *t*-test for continuous variables with normal distribution; Mann Whitney U test for continuous variables with non-normal distribution. Two-tailed p-value *a prior* set at 0.05

Abbreviations: AVC-H2, Avicenna's Hydrolyzed Chicken Collagen Type II; CI, confidence interval; SD, standard deviation; WOMAC, The Western Ontario and McMaster Universities Arthritis Index survey

Table S5. WOMAC sub-score in the difficulty in physical activities domain from baseline to follow-up weeks, after removing non-compliant participants.

Time point	Measurement [1]	Placebo [2] (mean ± SD)	AVC-H2 [2] (mean ± SD)	β Coefficient [3] (95% CI)	p-value [4]
Baseline	Raw score	19.0 ± 12.8	18.7 ± 11.7	---	0.903
Week 4	Raw score	16.3 ± 14.2	12.4 ± 10.8	---	0.184
	%Δ	-14.1 ± 60.1	-41.2 ± 37.7	-27.1 (-49.3, -4.9)	0.029
Week 8	Score	12.9 ± 13.7	10.3 ± 10.5	---	0.378
	%Δ	-33.8 ± 49.5	-48.2 ± 48.3	-14.5 (-36.8, 7.9)	0.211

[1] Raw score obtained from the WOMAC questionnaire; %Δ calculated as the difference between baseline and follow-up score, divided by baseline score, and presented in percentage format.

[2] Three participants removed from this set of analysis, 1 in the HC group and 2 in the placebo group, due to implausible %Δ value and incalculable %Δ (baseline score was 0 in this domain).

[3] β Coefficient and confidence intervals obtained from least squares regression analysis; the values were HC group minus placebo group.

[4] P-value was based on one of the following methods, where appropriate: Student's *t*-test for continuous variables with normal distribution; Mann Whitney U test for continuous variables with non-normal distribution. Two-tailed p-value *a prior* set at 0.05

Abbreviations: AVC-H2, Avicenna's Hydrolyzed Chicken Collagen Type II; CI, confidence interval; SD, standard deviation; WOMAC, The Western Ontario and McMaster Universities Arthritis Index survey

Table S6. VAS pain assessment scale from baseline to follow-up weeks, after removing non-compliant participants.

Time point	Measurement [1]	Placebo [2] (mean ± SD)	AVC-H2 [2] (mean ± SD)	β Coefficient [3] (95% CI)	p-value [4]
Baseline	Raw score	5.2 ± 1.4	5.3 ± 1.3	---	0.712
Week 4	Raw score	3.7 ± 2.2	2.8 ± 1.8	---	0.070
	%Δ	-30.6 ± 32.2	-43.9 ± 35.3	-13.3 (-28.6, 2.1) [5]	0.090
Week 8	Score	3.2 ± 2.3	2.2 ± 1.9	---	0.062
	%Δ	-39.8 ± 33.7	-54.6 ± 37.0	-14.8 (-30.9, 1.3) [6]	0.071

[1] Raw score obtained from the VAS scale; %Δ calculated as the difference between baseline and follow-up score, divided by baseline score, and presented in percentage format.

[2] One participant removed from this set of analysis in the HC group, due to implausible %Δ value.

[3] β Coefficient and confidence intervals obtained from least squares regression analysis; the values were HC group minus placebo group.

[4] P-value was based on one of the following methods, where appropriate: Student's *t*-test for continuous variables with normal distribution; Mann Whitney U test for continuous variables with non-normal distribution. Two-tailed p-value *a prior* set at 0.05

[5] Significant at 90%CI level (-26.3, -0.2).

[6] Significant at 90%CI level (-28.4, -1.1).

Abbreviations: AVC-H2, Avicenna's Hydrolyzed Chicken Collagen Type II; CI, confidence interval; SD, standard deviation; VAS, Visual Analogue Scale.
